# A Pan-Cancer Analysis of SMARCA4 Alterations in Human Cancers

**DOI:** 10.3389/fimmu.2021.762598

**Published:** 2021-10-04

**Authors:** Ling Peng, Jisheng Li, Jie Wu, Bin Xu, Zhiqiang Wang, Georgios Giamas, Justin Stebbing, Zhentao Yu

**Affiliations:** ^1^ Department of Respiratory Disease, Zhejiang Provincial People’s Hospital, Affiliated People’s Hospital, Hangzhou Medical College, Hangzhou, China; ^2^ Department of Medical Oncology, Qilu Hospital, Cheeloo College of Medicine, Shandong University, Jinan, China; ^3^ Cancer Center, Renmin Hospital of Wuhan University, Wuhan, China; ^4^ Department of Urology, Shouguang Hospital of Traditional Chinese Medicine, Shouguang, China; ^5^ Department of Biochemistry and Biomedicine, School of Life Sciences, University of Sussex, Brighton, United Kingdom; ^6^ Division of Cancer, Department of Surgery and Cancer, Imperial College London, London, United Kingdom; ^7^ Department of Thoracic Surgery, National Cancer Center, National Clinical Research Center for Cancer, Cancer Hospital and Shenzhen Hospital, Chinese Academy of Medical Sciences and Peking Union Medical College, Shenzhen, China

**Keywords:** SMARCA4, cancer, bioinformatics, immunity, oncogene, EMT

## Abstract

**Background:**

SMARCA4, the essential ATPase subunit of SWI/SNF chromatin remodeling complex, regulates transcription through the control of chromatin structure and is increasingly thought to play significant roles in human cancers. This study aims to explore the potential role of SMARCA4 with a view to providing insights on pathologic mechanisms implicated here.

**Methods:**

The potential roles of SMARCA4 in different tumors were explored based on The Cancer Genome Atlas (TCGA), Genotype-tissue expression (GTEx), Tumor Immune Estimation Resource (TIMER), and Gene Set Enrichment Analysis (GSEA) datasets. The expression difference, mutation and phosphorylation status, survival, pathological stage, DNA methylation, tumor mutation burden (TMB), microsatellite instability (MSI), mismatch repair (MMR), tumor microenvironment (TME), and immune cell infiltration related to SMARCA4 were analyzed.

**Results:**

High expression levels of SMARCA4 were observed in most cancer types. SMARCA4 expression in tumor samples correlates with poor overall survival in several cancers. Lung adenocarcinoma cases with altered SMARCA4 showed a poorer prognosis. Enhanced phosphorylation levels of S613, S695, S699, and S1417 were observed in several tumors, including breast cancer. SMARCA4 correlated with tumor immunity and associated with different immune cells and genes in different cancer types. TMB, MSI, MMR, and DNA methylation correlated with SMARCA4 dysregulation in cancers. SMARCA4 expression was negatively associated with CD8+ T-cell infiltration in several tumors. Furthermore, the SWI/SNF superfamily-type complex and ATPase complex may be involved in the functional mechanisms of SMARCA4, albeit these data require further confirmation.

**Conclusions:**

Our study offers a comprehensive understanding of the oncogenic roles of SMARCA4 across different tumors. SMARCA4 may correlate with tumor immunity.

## Introduction

The SMARCA subgroup of genes belong to the SWI1/SNF1 family and are responsible for chromatin remodeling and repair (1). SMARCA4 has been shown to be involved in developmental processes, transcriptional regulation, DNA repair, cell cycle control, and cancer (2). Inactivating mutations in SMARCA4 leads to loss of expression of protein within the nucleus and characterizes malignancies that are related, with SMARCA-deficiency. SMARCA4 was identified as a tumor suppressor gene; however, recent reports have revealed an oncogenic role of SMARCA4 (3). We conducted a pan-cancer genomic analysis of SMARACA4 across different cancer types using large-scale RNA-sequencing (RNA-seq) data from TCGA.

In the present study, through data mining analyses, we visualized the prognostic landscape of SMARCA4 expression and mutation across cancers and analyzed the expression of the SMARCA4 gene and its association with tumor-infiltrating immune cells (TIICs) and related immune markers. Our findings suggest that SMARCA4 influences the prognosis of cancer patients, while the role of SMARCA4 in different cancer types varies. Elevated *SMARCA4* gene expression is detrimental to survival in some situations with contradictory results in other tumor types.

Taking these facts together, these data suggest SMARCA4 is a prognostic marker for both clinical outcomes and immune infiltration.

## Material and Methods

### Gene Expression Analysis of SMARCA4

The “Gene_DE” module of tumor immune estimation resource, version 2 (TIMER2) web server (http://timer.cistrome.org/) was explored with input of “SMARCA4.” The differences of SMARCA4 gene expression between tumor and normal tissues of TCGA datasets were explored. For some tumors without normal tissues (e.g., TCGA-diffuse large B cell lymphomas (DLBC), TCGA-glioblastoma multiforme (GBM), TCGA-low-grade glioma (LGG), etc.), “Expression Analysis-Box Plots” module of the gene expression profiling interactive analysis, version 2 (GEPIA2) web server (http://gepia2.cancer-pku.cn/#analysis) was used to obtain expression difference between tumor and normal tissues of GTEx database. *p*-Value cutoff = 0.01, log2 fold change (FC) cutoff = 1, and “Match TCGA normal and GTEx data” were set as criteria. Violin plots of the SMARCA4 expression in different pathological stages of TCGA tumors through the “pathological stage plot” module of GEPIA2 were obtained. The log2 (transcripts per million (TPM) +1) transformed expression data were applied for the box or violin plots.

UALCAN portal (http://ualcan.path.uab.edu/analysis-prot.html) was used to perform protein expression analysis of the clinical proteomic tumor analysis consortium (CPTAC) dataset (4). Expression level of total protein or phosphoprotein of SMARCA4 between primary tumor and normal tissues were explored. Datasets of six tumors were selected, including breast cancer, ovarian cancer, colon cancer, clear cell renal cell carcinoma (RCC), uterine corpus endometrial carcinoma (UCEC), and lung adenocarcinoma (LUAD).

### Survival Prognosis of SMARCA4

Survival map of SMARCA4 across TCGA tumors were generated from the “Survival Map” module of GEPIA2, in terms of overall survival (OS) and disease-free survival (DFS). Cutoff value of 50% was set as expression threshold for separating high- and low-expression cohorts. The log-rank test was used in the hypothesis test, and the survival plots were generated *via* “survival analysis” module of GEPIA2.

### Genetic Alteration Analysis of SMARCA4

The cBioPortal website (https://www.cbioportal.org/) was explored using “quick selection” section to investigate “TCGA Pan Cancer Atlas Studies”. “SMARCA4” was entered for queries of the genetic alteration. Alteration frequency, mutation type, and copy number alteration (CNA) results of all TCGA tumors were obtained from “cancer types summary” module. The “mutations” module was used to explore the mutated site of SMARCA4, which is displayed in the schematic diagram of the protein structure or the three-dimensional (3D) structure. The “comparison” module was used to generate the data on the overall, disease-free, progression-free, and disease-free survival of TCGA cases with or without SMARCA4 alteration. Kaplan-Meier plots with log-rank *p*-value were displayed.

### Immune Infiltration Analysis of SMARCA4

We first evaluated the relationship between the level of SMARCA4 expression and the abundance of six types of TIICs, including CD4+ T cells, CD8+ T cells, B cells, neutrophils, dendritic cells (DCs), and macrophages. Results were exhibited in the form of these three kinds of scores: ImmuneScore, StromalScore, and ESTIMATEScore. The higher score estimated in ImmuneScore or StromalScore positively correlated with the ratio of immune or stromal, and it referred to the higher the respective score and the larger the ratio of the corresponding component in TME. ESTIMATEScore was the sum of both, denoting the integrated proportion of both components in TME. The ImmuneScore and StromalScore of multiple cancers were obtained *via* the “estimate” R package.

The purity of tumors was also quantified, and the differences of 22 immune cell subtypes were further explored using the “Immune-Gene” module of TIMER2 web server. The algorithms of TIMER, CIBERSORT, CIBERSORT-ABS, QUANTISEQ, XCELL, MCPCOUNTER, and EPIC were used to estimate immune infiltration. The *p*-values and partial correlation values (cor) were obtained *via* the purity-adjusted Spearman’s rank correlation test. The ratio of immune-stromal component in tumor microenvironment (TME) to obtained explores the association of the estimated proportion of immune and stromal with SMARCA4 expression using Spearman’s correlation analysis.

Spearman’s correlation analysis was also used to evaluate the relationships between SMARCA4 expression and expression levels of the immune checkpoint markers. Gene markers of TIICs were analyzed including T cells (general), CD8+ T cells, B cells, monocytes, TAMs, M1 macrophages, M2 macrophages, DCs, neutrophils, natural killer (NK) cells, follicular helper T (Tfh) cells, T-helper 1 (Th1) cells, T-helper 2 (Th2) cells, T-helper 17 (Th17) cells, exhausted T cells, Tregs, and mast cells (5). These gene markers include BLTA, CD200, TNFRSF14, NRP1, LAIR1, TNFSF4, CD244, LAG3, ICOS, CD40LG, CTLA4, CD48, CD28, CD200R1, HAVCR2, ADORA2A, CD276, KIR3DL1, CD80, PDCD1, LGALS9, CD160, TNFSF14, IDO2, ICOSLG, TMIGD2, VTCN1, IDO1, PDCD1LG2, HHLA2, TNFSF18, BTNL2, CD70, TNFSF9, TNFRSF8, CD27, TNFRSF25, VSIR, TNFRSF4, CD40, TNFRSF18, TNFSF15, TIGIT, CD274, CD86, CD44, and TNFRSF9. All of the gene expression levels were log2 transformed.

Relationship between SMARCA4 and TMB or MSI was analyzed. The “maftools” R package was applied to analyze the somatic data (MAF data) in human pan-cancer from the TCGA database. The number of mutations of exons to identify the TMB was analyzed in each cancer. MSI score was obtained from the TCGA database. The analysis of the association between SMARCA4 expression and TMB or MSI was based on the Spearman’s method.

### Analysis of SMARCA4 Expression and MMR Gene Mutation and DNA Methylation

MLH1, MSH2, MSH6, EPCAM, and PMS2 are five MMR genes, and their expression levels in multiple cancers were obtained from the TCGA database. The correlation of expression levels of these MMR genes with the expression levels of SMARCA4 was analyzed by the Spearman’s correlation method. In the present study, we also evaluated the expression levels of DNMT1, DNMT2, DNMT3A, and DNMT3B, and Spearman’s correlation was used to evaluate the correlation of the four methyltransferases with SMARCA4 expression.

### Gene-Related Enrichment Analysis

The STRING website (https://string-db.org/) was used to query protein name “BRG1” and organism (“*Homo sapiens*”). The following main parameters were set as: minimum required interaction score [“low confidence (0.150)”], meaning of network edges (“evidence”), max number of interactors to show (“no more than 50 interactors” in 1st shell) and active interaction sources (“experiments”). Then, the available experimentally determined SMARCA4-binding proteins were obtained. The “similar gene detection” module of GEPIA2 was used to obtain the top 100 SMARCA4-correlated targeting genes of all TCGA tumor and normal tissues. The “correlation analysis” module of GEPIA2 was used to perform a pairwise gene Pearson’s correlation analysis of SMARCA4 and selected genes. The log2 TPM was applied for the dot plot. The *p*-value and the correlation coefficient (*R*) were indicated. The “Gene_Corr” module of TIMER2 was used to generate heatmap data of the selected genes, which contains the partial correlation (cor) and *p*-value in the purity-adjusted Spearman’s rank correlation test. Venn diagram was used to conduct an intersection analysis to compare the SMARCA4-binding and interacted genes.

Two sets of data were combined to perform Kyoto Encyclopedia of Genes and Genomes (KEGG) pathway analysis. The gene lists were uploaded to Database for Annotation, Visualization, and Integrated Discovery (DAVID) with the settings of selected identifier (“OFFICIAL_GENE_SYMBOL”) and species (“*Homo sapiens*”) and obtained the data of the functional annotation chart. Enriched pathways were visualized with R packages “tidyr” and “ggplot2.” Gene Ontology (GO) enrichment analysis was conducted using R package “clusterProfiler”. The data for biological process (BP), cellular component (CC), and molecular function (MF) were visualized as cnet plots, using the cnetplot function.

GSEA was performed in the high- and the low-expression groups to explore the biological signaling pathway. The top 5 terms of KEGG and HALLMARK analyses were exhibited. KEGG pathways with significant enrichment results were demonstrated on the basis of net enrichment score (NES), gene ratio, and *p*-value. Gene sets with |NES| >, NOM *p* **<** 0.05, and FDR *q* < 0.25 were considered to be enrichment significant (6).

### Statistical Analysis

R language software (version 4.0.3) (https://www.r-project.org/) was used in this analysis. Differences between the two groups and among multiple groups were analyzed using the default Wilcoxon’s test and one-way analysis of variance (ANOVA), respectively. The differences in overall survival between groups were determined by Kaplan-Meier analysis and a log-rank test. The subtypes, clinicopathological features, risk scores, expression of immune checkpoints, and levels of immune infiltration were determined by a Pearson’s correlation test. Results were considered statistically significant when the *p* **<** 0.05.

## Results

### Analysis of Gene Expression

**Figure 1:**
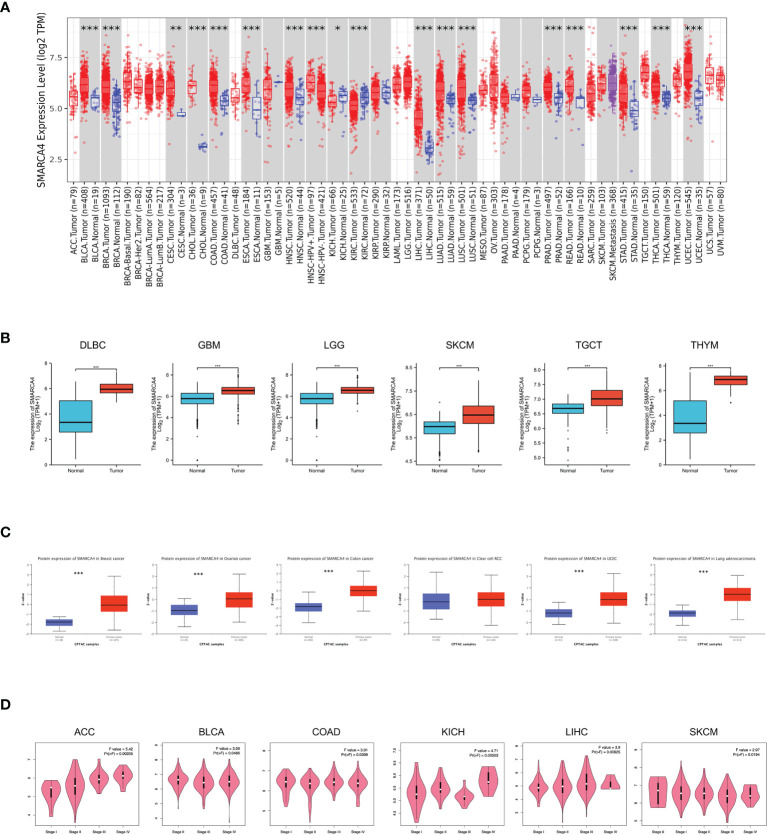
Expression level of *SMARCA4* gene in different tumors and pathological stages. **(A)** The expression status of the *SMARCA4* gene in different cancers or specific cancer subtypes was analyzed through TIMER2. *p < 0.05; **p < 0.01; ***p < 0.001. **(B)** For the type of DLBC, GBM, LGG, SKCM, TGCT, and THYM in the TCGA project, the corresponding normal tissues of the GTEx database were included as controls. The box plot data were supplied. **p < 0.01. **(C)** Based on the CPTAC dataset, we also analyzed the expression level of SMARCA4 total protein between normal tissue and primary tissue of breast cancer, ovarian cancer, colon cancer, clear cell RCC, and UCEC. ***p < 0.001. **(D)** Based on the TCGA data, the expression levels of the *SMARCA4* gene were analyzed by the main pathological stages (stages I, II, III, and IV) of ACC, BLCA, COAD, KICH, LIHC, and SKCM. Log2 (TPM +1) was applied for log scale.

The expression level of SMARCA4 in tumor tissues was higher than the corresponding control tissues, including bladder urothelial carcinoma (BLCA), breast invasive carcinoma (BRCA), cholangiocarcinoma (CHOL), colon adenocarcinoma (COAD), esophageal carcinoma (ESCA), head and neck squamous cell carcinoma (HNSC), liver hepatocellular carcinoma (LIHC), lung adenocarcinoma (LUAD), lung squamous cell carcinoma (LUSC), prostate adenocarcinoma (PRAD), rectum adenocarcinoma (READ), stomach adenocarcinoma (STAD), thyroid carcinoma (THCA), UCEC (all *p* **<** 0.001) and cervical squamous cell carcinoma and endocervical adenocarcinoma (CESC, *p* **<** 0.01), as shown in **Figure 1A**. The expression level of SMARCA4 in tumor tissues was significantly lower than control tissues of kidney renal clear cell carcinoma (KIRC, *p* **<** 0.001) and kidney chromophobe (KICH, *p* **<** 0.05).

The expression differences of SMARCA4 between the tumor and normal tissues of lymphoid neoplasm diffuse large B-cell lymphoma (DLBC), glioblastoma multiforme (GBM), brain lower grade glioma (LGG), skin cutaneous melanoma (SKCM), testicular germ cell tumors (TGCT), and thymoma (THYM) were also analyzed in GTEx dataset (**Figure 1B**, *p* **<** 0.001). The results of the CPTAC dataset showed higher expression of SMARCA4 total protein in the primary tissues of breast cancer, ovarian cancer, colon cancer, UCEC and LUAD (**Figure 1C**, *p* **<** 0.001) than in normal tissues.

We also used the “pathological stage plot” module of GEPIA2 to observe the correlation between SMARCA4 expression and the pathological stages of cancers, including adrenocortical carcinoma (ACC), BLCA, CESC, COAD, KICH, LUAD, pancreatic adenocarcinoma (PAAD), and THCA (**Figure 1D**, all *p* **<** 0.05).

### Analysis of Survival Prognosis

**Figure 2:**
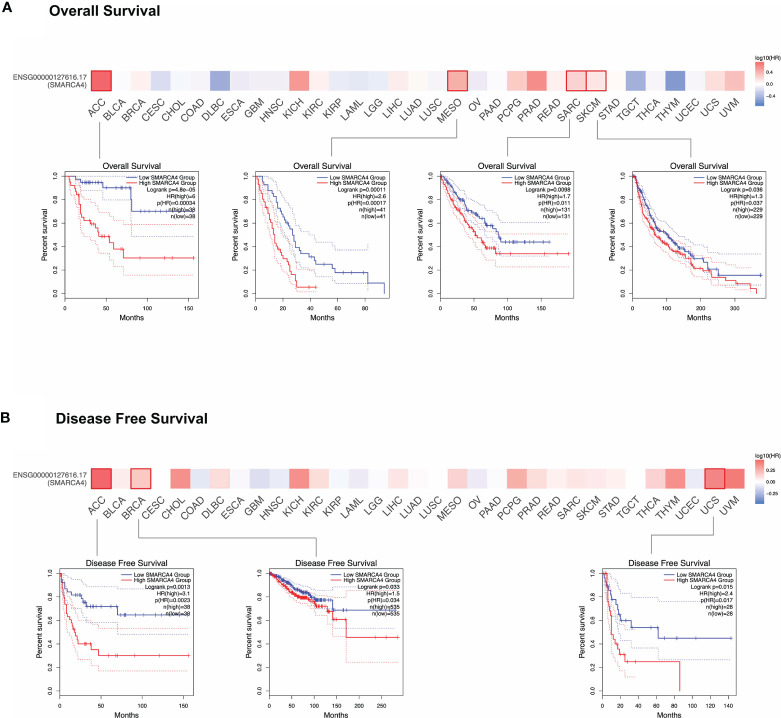
Correlation between *SMARCA4* gene expression and survival prognosis of cancers in TCGA. GEPIA2 tool was used to perform overall survival **(A)** and disease-free survival **(B)** analyses of different tumors in TCGA by *SMARCA4* gene expression. The survival map and Kaplan-Meier curves with positive results are given.

As shown in **Figure 2A**, highly expressed SMARCA4 is linked to poor prognosis for cancers including ACC (*p* = 0.00034), mesothelioma (MESO, *p* = 0.00017), sarcoma (SARC, *p* = 0.011), and SKCM (*p* = 0.037) of TCGA datasets. DFS analysis (**Figure 2B**) shows high SMARCA4 expression is correlated with poor prognosis for the TCGA cases of ACC (*p* = 0.0023), BRCA (*p* = 0.034), and uterine carcinosarcoma (UCS, *p* = 0.017). The above data indicate that SMARCA4 expression is associated with the prognosis of cases with different tumors.

### Genetic Alteration Analysis

**Figure 3:**
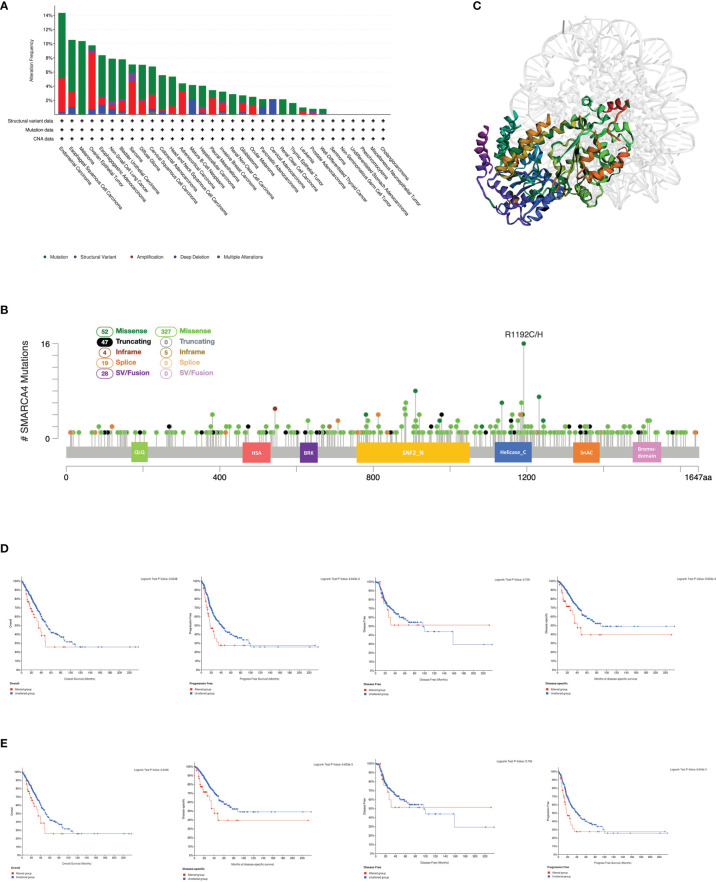
Mutation feature of SMARCA4 in different tumors of TCGA. We analyzed the mutation features of SMARCA4 for the TCGA tumors using the cBioPortal tool. **(A)** The alteration frequency with mutation type is displayed. **(B)** Mutation sites are displayed. **(C)** The 3D structure of SMARCA4 was displayed. **(D)** The potential correlation between mutation status and overall, disease-specific, disease-free, and progression-free survival of UCEC using the cBioPortal tool. **(E)** The potential correlation between mutation status and overall, disease-specific, disease-free, and progression-free survival of LUAD using the cBioPortal tool.

The genetic alteration status of SMARCA4 in different tumor samples of the TCGA cohorts were analyzed. As shown in **Figure 3A**, the highest alteration frequency of SMARCA4 appears for patients with uterine tumors with “mutation” as the primary type.

The “amplification” type of CNA is the primary type in the ovarian cancer cases, which shows an alteration frequency of ~9% (**Figure 3A**). It is worth noting that all kidney cases (clear cell and nonclear cell carcinoma) with genetic alteration have SMARCA4 mutations. The types, sites, and case number of the SMARCA4 genetic alterations are further presented in **Figure 3B**. Missense mutation of SMARCA4 is the main type of genetic alteration. The R1192C/H alteration in the helicase_C domain is detected in 16 cases (**Figure 3B**). The 3D structure of SMARCA4 protein is shown in **Figure 3C**. The potential association between SMARCA4 alteration and the clinical survival prognosis of cases with different types of cancer was analyzed. The data in **Figure 3D** indicate that UCEC cases with altered SMARCA4 have better prognosis in PFS (*p* = 0.0387), but not OS (*p* = 0.158), DFS (*p* = 0.0762), and disease-specific survival (DSS) (*p* = 0.147), compared with patients without SMARCA4 alterations. Interestingly, the data of **Figure 3E** indicate that LUAD cases with altered SMARCA4 show worse prognosis in OS (*p* = 0.0348), PFS (*p* = 0.0387), DFS (*p* = 0.0762), and DSS (*p* = 0.147), compared with patients without SMARCA4 alterations.

### Analysis of Protein Phosphorylation

**Figure 4:**
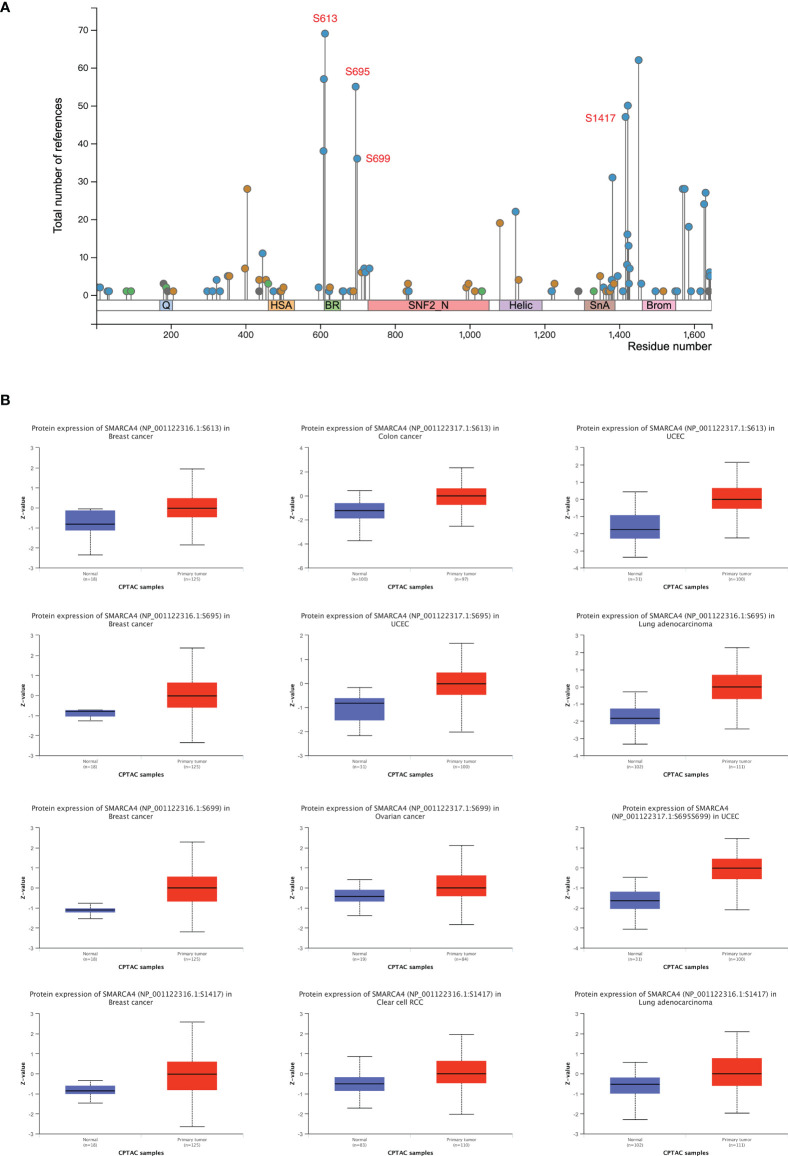
Phosphorylation analysis of SMARCA4 in different tumors. Based on the CPTAC dataset, the expression level of SMARCA4 phosphoprotein was analyzed between normal tissue and primary tissue of selected tumors *via* the UALCAN. **(A)** The phosphoprotein sites with positive results are displayed in the schematic diagram of SMARCA4 protein. **(B)** The box plots for different cancers are displayed.

The SMARCA4/BRG1 phosphorylation levels between tumor *versus* normal tissues of five types of tumors (breast cancer, clear cell RCC, LUAD, ovarian cancer, and UCEC) were analyzed using CPTAC dataset. SMARCA4 phosphorylation sites and the significant differences are summarized in **Figure 4A**.

The S613 locus of SMARCA4 exhibits a higher phosphorylation level in several tumor tissues compared with normal tissues, including breast cancer, colon cancer and UCEC (**Figure 4B**, all *p* **<** 0.05), followed by an increased phosphorylation level of the S695 locus for breast cancer (*p* = 3.65*e*−06), UCEC (*p* = 2.34*e*−09), and LUAD (*p* = 5.72*e*−33). Higher phosphorylation level in some tumor tissues were also observed in S699 and S1417 locus.

### Immune Infiltration Analysis

**Figure 5:**
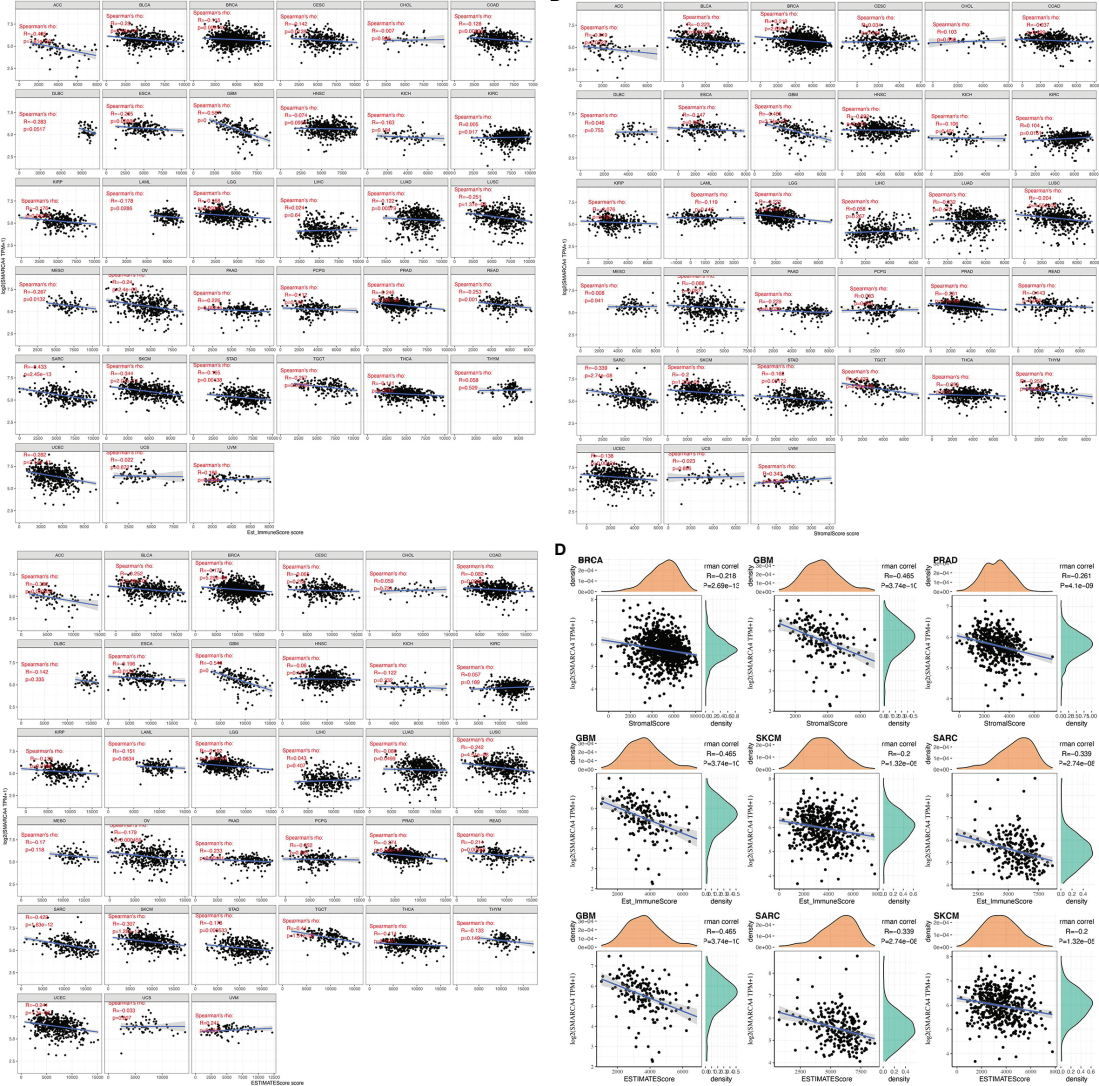
Correlation of SMARCA4 expression with ImmuneScore, StromalScore, and ESTIMATEScore in various cancers. **(A)** Correlation of SMARCA4 expression with ImmuneScore. **(B)** Correlation of SMARCA4 expression with StromalScore. **(C)** Correlation of SMARCA4 expression with ESTIMATEScore. **(D)** Top 3 cancers by ImmuneScore, StromalScore, and ESTIMATEScore, respectively.

ImmuneScore and StromalScore were integrated to evaluate the relationship between SMARCA4 expression and immune infiltration across cancers. According to the results, SMARCA4 expression was negatively correlated with the ImmuneScore in ACC, BLCA, BRCA, etc. (**Figure 5A**). Also, SMARCA4 expression positively correlates with the StromalScore in UVM, while negatively in ACC, BLCA, BRCA, COAD, etc. (**Figure 5B**). SMARCA4 expression is negatively correlated with ESTIMATEScore in most cancer types (**Figure 5C**). The top 3 tumors most significantly correlated with expression of SMARCA4 are BRCA, GBM, and PRAD (StromalScore); GBM, SKCM, and SARC (ImmuneScore); and GBM, SARC, and SKCM (ESTIMATEScore) (**Figure 5D**).

**Figure 6:**
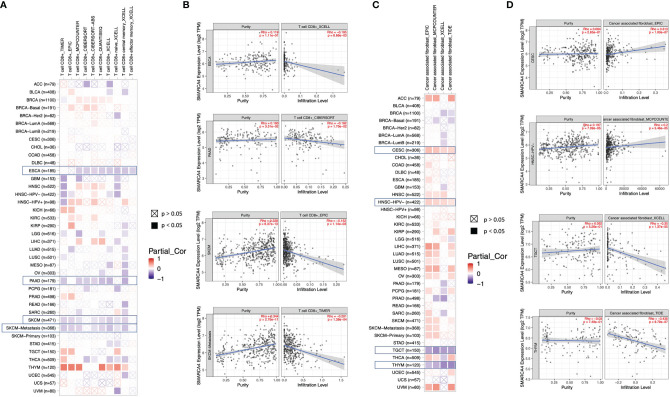
Correlation analysis between SMARCA4 expression and immune infiltration of CD8+ T cell and cancer-associated fibroblasts base on analysis of 22 immune cell types. Different algorithms were used to explore the potential correlation between the expression level of the SMARCA4 gene and the infiltration level of **(A, B)** CD8+ T cell and **(C, D)** cancer-associated fibroblasts across all types of cancer in TCGA.

Algorithms of TIMER, CIBERSORT, CIBERSORT-ABS, QUANTISEQ, XCELL, MCPCOUNTER, and EPIC were further used to investigate the potential relationship between the infiltration level of different immune cells and *SMARCA4* gene expression in diverse cancer types of TCGA. A statistically negative correlation was observed between the immune infiltration of CD8^+^ T cells and SMARCA4 expression in the tumors of ESCA, PAAD, SKCM, and SKCM-metastasis (**Figure 6A**) based on most algorithms. For example, SMARCA4 expression level in PRAD is negatively correlated with the infiltration level of CD8+ T cells (**Figure 6B**, cor = −0.152, *p* = 1.14*e*−03) based on the EPIC algorithm. A statistically positive correlation of SMARCA4 expression and the estimated infiltration value of cancer-associated fibroblasts is observed for the TCGA tumors of CESC and HNSC-HPV− but noted a negative correlation for TGCT and THYM (**Figure 6C**). For example, SMARCA4 expression level in HNSC-HPV− is correlated with the infiltration level of cancer-associated fibroblasts (**Figure 6D**, cor = 0.2, *p* = 5.46*e*−05) based on the MCPCOUNTER algorithm.

**Figure 7:**
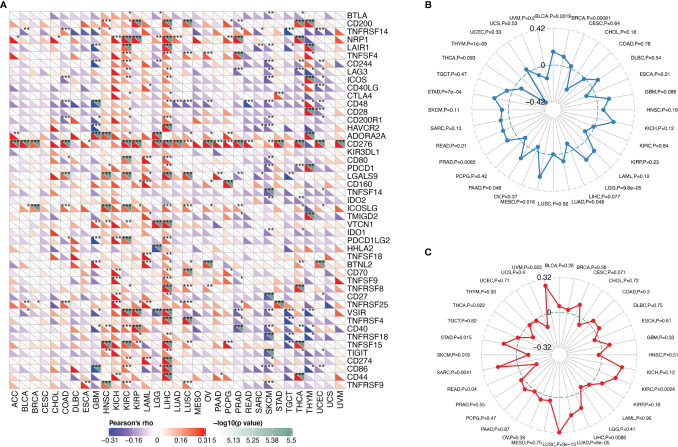
Correlations between SMARCA4 expression and immunity, including immune marker sets, TMB, and MSI in cancers. **(A)** Correlation between SMARCA4 expression and immune marker sets. **(B)** Radar map of correlation between SMARCA4 expression and TMB. The value in black reveals the range, and the curve in blue reveals the correlation coefficient. **(C)** Radar map of correlation between SMARCA4 expression and MSI. The value in black reveals the range, and the curve in red reveals the correlation coefficient. *p < 0.05, **p < 0.01, ***p < 0.001.

The correlation between SMARCA4 and immune checkpoint gene expression was analyzed. In LIHC, SMARCA4 expression is positively correlated with expression of CD200, NRP1, LAIR1, TNFSF4, LAG3, ICOS, CD48, CD200R1, HAVCR2, CD276, CD80, PDCD1, LGALS9, ICOSLG, TIGIT, etc. (**Figure 7A**).

Association between SMARCA4 expression and TMB varies markedly among cancer types. SMARCA4 is positively correlated with TMB in BLCA, KICH, LIHC, LUAD, MESO, etc. (**Figure 7B**). On the contrary, SMARCA4 expression is negatively associated with TMB in UVM, THYM, CHOL, etc. SMARCA4 is positively correlated with MSI in UVM, KICH, SARC, STAD, LUSC, LUAD, etc. (**Figure 7C**). In contrast, SMARCA4 expression is negatively related to MSI in CHOL, READ, SKCM, UCS, etc. All these data together indicate that high SMARCA4 expression is widely associated with immunity in cancers.

### SMARCA4 Is Associated With MMR Gene and DNA Methylation

**Figure 8:**
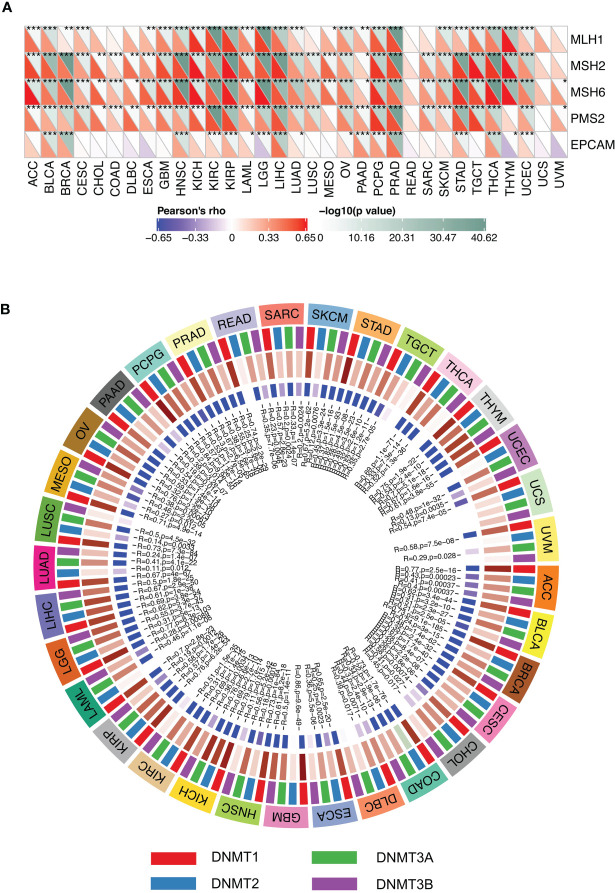
Expression levels of SMARCA4 correlate with five MMR genes and four DNA methyltransferases. **(A)** Spearman’s correlation analysis of SMARCA4 expression with expression levels of five MMR genes across cancers (*p < 0.05, **p < 0.01, ***p < 0.001). **(B)** Spearman’s correlation analysis of SMARCA4 expression with four DNA methyltransferases across cancers.

In order to determine the potential role of SMARCA4 in tumor progression, we evaluated the association of the expression level of SMARCA4 with mutation levels of five MMR genes. The results shown in **Figure 8A** revealed that SMARCA4 is highly related to MMR genes in 31 cancers, except for READ and UCS. The relationships between SMARCA4 and four DNA methyltransferases were also evaluated. SMARCA4 expression is highly associated with these four DNA methyltransferases in multiple cancers, such as THYM, BLCA, COAD, etc. (**Figure 8B**). These results indicate that SMARCA4 may regulate the tumor progression by mediating repairment of DNA and DNA methylation across cancers.

### Enrichment of SMARCA4-Related Partners

**Figure 9:**
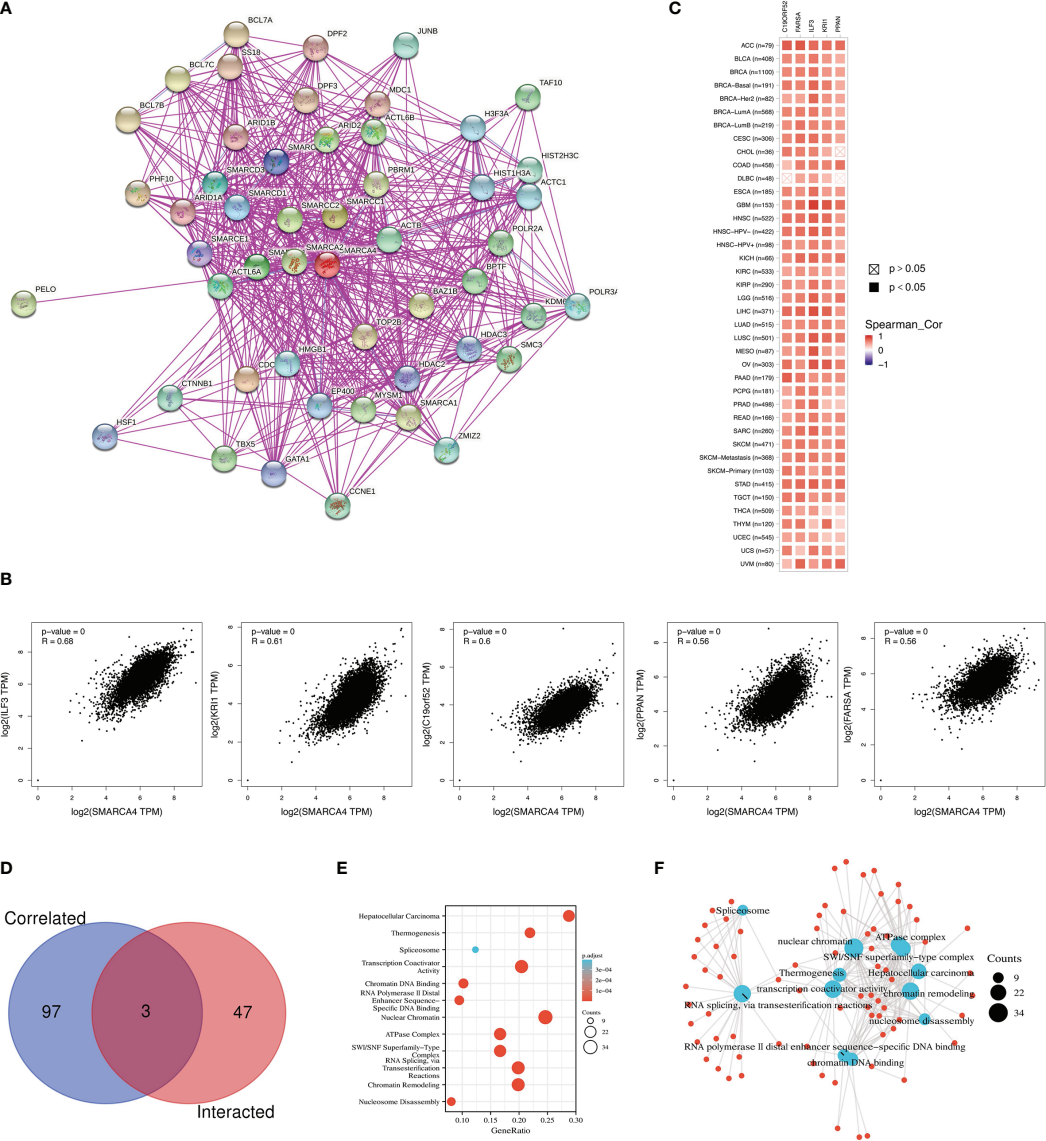
SMARCA4-related gene enrichment analysis. **(A)** The available experimentally determined SMARCA4-binding proteins using the STRING tool were obtained. **(B)** The top 100 SMARCA4-correlated genes in TCGA were generated using GEPIA2, and the expression correlation between SMARCA4 and selected targeting genes were analyzed. **(C)** Heatmap data in the detailed cancer types are displayed. **(D)** An intersection analysis of the SMARCA4-binding and correlated genes was conducted. **(E)** KEGG pathway analysis based on the SMARCA4-binding and interacted genes. **(F)** The cnetplot for the molecular function data in GO analysis.

A total of 50 SMARCA4-binding proteins were obtained which were supported by experimental evidence *via* STRING tool. Interaction network of these proteins is shown in **Figure 9A**. The top 100 genes that correlate with SMARCA4 expression were obtained *via* GEPIA2 tool to combine pan-cancer expression of TCGA. As shown in **Figure 9B**, SMARCA4 expression level is positively correlated with that of interleukin enhancer-binding factor 3 (IL-F3) (*R* = 0.68), Krev interaction trapped/cerebral cavernous malformation 1 (KRI1) (*R* = 0.61), C19orf52 (TIMM29) (*R* = 0.6), Peter Pan Homolog (PPAN) (*R* = 0.56), and phenylalanyl-tRNA synthetase alpha (FARSA) (*R* = 0.56) genes (all *p* **<** 0.001). The corresponding heatmap data also show a positive correlation between SMARCA4 and the above five genes in the majority of detailed cancer types (**Figure 9C**). Intersection analysis of the above two groups show three common members, namely, SMARCB1, SMARCD1, and HDAC2 (**Figure 9D**).

We combined the two datasets to perform KEGG and GO enrichment analyses. KEGG data suggest that “SWI/SNF superfamily-type complex” and “ATPase complex” might be involved in the effect of SMARCA4 on tumor pathogenesis, which was shown in **Figure 9E**. GO enrichment analysis indicate that most of these genes are linked to the pathways or cellular biology of chromatin, such as chromatin remodeling, chromatin DNA binding, nuclear chromatin, and others (**Figure 9F**).

**Figure 10:**
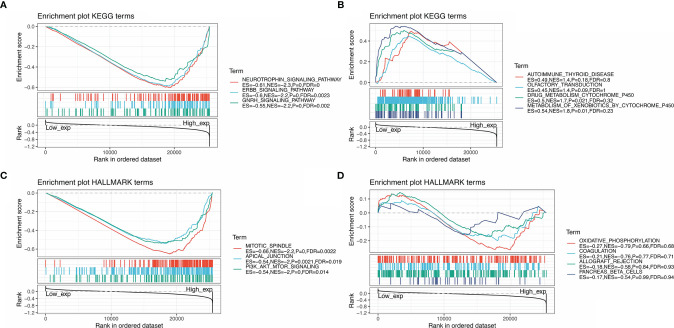
GSEA for samples with high SMARCA4 expression and low expression. **(A)** The enriched gene sets in KEGG collection by the high SMARCA4 expression sample. **(B)** The enriched gene sets in KEGG by samples with low SMARCA4 expression. **(C)** Enriched gene sets in HALLMARK collection, the immunologic gene sets, by samples of high SMARCA4 expression. **(D)** Enriched gene sets in HALLMARK by the low SMARCA4 expression. Each line representing one particular gene set with unique color, and upregulated genes located in the left approaching the origin of the coordinates, by contrast the downregulated lay on the right of *x*-axis. Only gene sets with NOM *p* < 0.05 and FDR *q* < 0.06 were considered significant. Only several leading gene sets were displayed in the plot.

GSEA was performed to identify the functional enrichment of high and low SMARCA4 expression (**Figure 10**). KEGG enrichment term exhibits that high expression of SMARCA4 is mainly associated with tyrosine kinase signaling pathways, including neurotrophin signaling pathway, ERBB signaling pathway, and GnRH pathway. HALLMARK terms indicated that high expression of SMARCA4 was associated with mitotic spindle, apical junction, and PI-3K/AKT/mTOR signaling pathways.

## Discussion

The chromatin remodeling switch sucrose nonfermentable SWI/SNF complex is evolutionarily conserved and comprised of a catalytic subunit, either of BRG1 (also known as SMARCA4) or of BRM (also known as SMARCA2) (7). The SWI/SNF complex is involved in the pathogenesis cancer of several organs with therapeutic and prognostic value (8). SMARCA4, a key SWI/SNF chromatin remodeling gene, is frequently inactivated in cancers and is not directly druggable (9). SMARCA4 is regarded as a *bona fide* tumor suppressor and cooperates with p53 loss and Kras activation (10). Studies reveal that about 20% of human cancers contain mutations in SWI/SNF enzyme subunits, indicating that the enzyme subunits are critical tumor suppressors (11). On the other hand, the oncogenic role of SMARCA4 has been reported in several cancer types (12, 13). The role of SMARCA4 across cancers and whether it can serve as a prognostic biomarker remain to be determined. Therefore, the pan-cancer analysis of SMARCA4 is vital and useful for comparing the similarities and differences among different cancers.

The present work illustrated a comprehensive workflow for pan-cancer analysis and thoroughly investigated the role of the SMARCA4 in cancers. The results show that overexpression of SMARCA4 is correlated with a worse prognosis in some cancer types. SMARCA4 expression is closely associated with the levels of immune infiltration. Furthermore, SMARCA4 is aberrantly expressed in various cancers and significantly associated with MMR, MSI, DNA methylation, and TMB. Therefore, SMARCA4 may play an essential role in cancer prognosis and tumor immunity.

It was reported that loss of SWI/SNF protein expression in NSCLC is associated with aggressive clinicopathological features, PD-L1-positive status, and high TMB in NSCLC (14). Studies have also revealed that SMARCA4 expression was associated with poor prognosis of multiple cancer types, including liver hepatocellular carcinoma and kidney renal clear cell carcinoma (15), challenging the role of SMARCA4 as a tumor suppressor. While mammalian SWI/SNF enzyme function is highly context dependent and the enzymatic activity generates changes in chromatin accessibility, which can either negatively or positively affect chromatin utilization, therefore, overexpression of SMARCA4 may similarly cause initiation or acceleration of cancer, which is not unexpected (11).

SMARCA4 alterations can be divided into two clinically relevant genomic classes associated with differential protein expression as well as distinct prognostic and treatment implications (8). As loss of BRG1 expression requires bi-allelic inactivation, in NSCLCs with truncating SMARCA4 mutations, nearly half of these mutations resulted in BRG1 deficiency (16). SMARCA4 RNA splicing defects, expression of particular microRNAs, signaling pathway activation such as PI3K or AKT have been shown to downregulate SMARCA4 expression in lung cancer, therefore, screening strategies that exclusively rely on next-generation sequencing may fail to detect lung cancers with nonmutational mechanisms of BRG1 inactivation (16).

Mutations of SMARCA4 represent a genetic factor leading to adverse clinical outcome in lung adenocarcinoma (17). SMARCA4-mutant lung cancers may be more sensitive to immunotherapy (8), but other studies have contradictory results (18). The conflicting results on the role of SMARCA4 might be due to several reasons such as limited sample size, different treatment, and co-occurring mutations. Co-occurring mutations exist, such as STK11/KEAP1, and these genes were identified to be potentially associated with reduced efficacy of immunotherapy (KEAP1, PBRM1, SMARCA4, and STK11) (19). The discovery that the SMARCA4 plays an essential role in cancer immunology highlights the importance of future studies of larger cohorts of patients to further determine the clinical feasibility of immune checkpoint inhibitors.

SMARCA4 expression is negatively correlated with ESTIMATEScore in most cancer types. ESTIMATEScore indicates the purity of the tumor (5), and low purity suggests advanced stage and poor prognosis in cancer (20). In addition, SMARCA4 is positively correlated with expression of multiple immune checkpoint genes, and SMARCA4 expression is related to high TMB in some cancer types, such as lung adenocarcinoma. This result is consistent with that reported by Foundation Medicine (16) and other reports (20). These results indicate that SMARCA4 is involved intensely with tumor immune evasion. Furthermore, immune infiltration analysis of SMARCA4 reveal a negative correlation CD8+ T cells and SMARCA4 expression in the tumors of ESCA, PAAD, SKCM, and SKCM-metastasis, while the correlation of SMARCA4 and CD8+ T cells in other tumor types still needs further investigation.

The correlation of SMARCA4 with MSI in various cancer types was also investigated in our study. MMR genes play a vital role in maintaining the stability of the genome. In our analysis, SMARCA4 expression is related with the expression of MMR genes in multiple cancer types. We found that SMARCA4 expression is highly related to five MMR genes and to MSI in most cancer types. Previous studies also found SWI/SNF-mutated gastric cancer are correlated with MSI genotype (21). DNA methylation is an epigenetic mechanism and a novel predictor for tumorigenesis. SMARCA4 is a novel key epigenetic modulator of colorectal cancer (22), and SMARCA4 may directly influence the loss of DNA methylation, which provided insight of aberrant gene induction during tumor progression (23).

The phosphorylation findings from CPTAC dataset including six cancer types indicated that enhanced phosphorylation levels of S613, S695, S699, and S1417 were observed in several tumors. Whether these posttranslational modification sites have clinical significance remains to be determined. We also analyzed the key signaling pathways of SMARCA4. The results indicate that SMARCA4 is related with DNA repair pathway. SMARCA4 enhances sensitivity to drugs that target oxidative phosphorylation and inhibit SMARCA2, EZH2, CDK4, or CDK6 (16). BET inhibitor has also emerged as a promising drug for the treatment of SMARCA4-deficient hepatocellular carcinoma based on preclinical studies (24), indicating that this particular subtype of cancer patients may benefit from precision targeted therapy.

Our study has several limitations. Firstly, the mRNA level of SMARCA4 is assessed in our study, while its correlation with protein levels need to be validated in future studies. Secondly, additional validation in other public datasets is required to further support our present findings. Thirdly, as multiple information from diverse databases was retrieved for the analysis, systematic bias exists. More efforts are needed to explore the role of SMARCA4 in cancer and the value of SMARCA4 as a potential target of anticancer therapy.

Taken together, our first pan-cancer analysis of SMARCA4 indicated clinically significant correlation of SMARCA4 with prognosis, DNA methylation, protein phosphorylation, immune cell infiltration, and immunity markers as TMB and MSI, which help understand the role of SMARCA4 in tumorigenesis.

## Conclusions

In summary, the present study reveals that SMARCA4 is correlated with the prognosis of patients with cancer and immune infiltration across diverse cancers. SMARCA4 gene expression is associated with MMR, MSI, TMB, and DNA methylation in multiple cancers. *SMARCA4* gene expression was strongly associated with the gene expression of immunity in various cancers. SMARCA4 may play a key role as a prognostic biomarker.

## Data Availability Statement

The datasets presented in this study can be found in online repositories. The names of the repository/repositories and accession number(s) can be found in the article/supplementary material.

## Author Contributions

LP, BX, and JL designed and supervised the study. LP, JL, JW, ZW, and BX analyzed the data and wrote the original draft. LP, JS, and GG edited the draft. All authors contributed to the article and approved the submitted version.

## Funding

This study was partially supported by the Natural Science Foundation of Zhejiang Province, China (Grant Number: LY19H160041).

## Conflict of Interest

JS, the Editor-in-Chief of Oncogene has sat on SABs for Vaccitech, Heat Biologics, Eli Lilly, Alveo Technologies, Pear Bio, Agenus, Equilibre Biopharmaceuticals, Graviton Bioscience Corporation, Celltrion, Volvox, Certis Oncology Solutions, Greenmantle, Zedsen, Bryologyx, and Benevolent AI. He has consulted with Lansdowne partners and Vitruvian. He sits on the Board of Directors for Xerion and BB Biotech Healthcare Trust PLC. GG is Editor in Chief in Cancer Gene Therapy and the Founder and Chief Scientific Advisor of Stingray Bio. None are relevant here.

The remaining authors declare that the research was conducted in the absence of any commercial or financial relationships that could be construed as a potential conflict of interest.

## Publisher’s Note

All claims expressed in this article are solely those of the authors and do not necessarily represent those of their affiliated organizations, or those of the publisher, the editors and the reviewers. Any product that may be evaluated in this article, or claim that may be made by its manufacturer, is not guaranteed or endorsed by the publisher.
